# Factors associated with minimum dietary diversity failure among Indian children

**DOI:** 10.1017/jns.2022.2

**Published:** 2022-02-04

**Authors:** Rajesh Kumar Rai, Sandhya S. Kumar, Chandan Kumar

**Affiliations:** 1 Society for Health and Demographic Surveillance, Suri 731101, West Bengal, India; 2 Department of Economics, University of Göttingen, Göttingen 37073, Germany; 3 Centre for Modern Indian Studies, University of Göttingen, Göttingen 37073, Germany; 4 Department of Global Health and Population, Harvard T H Chan School of Public Health, Boston, MA 02115, USA; 5 World Vegetable Center - South and Central Asia, Hyderabad 502324, Telangana, India; 6 Department of Policy and Management Studies, TERI School of Advanced Studies, New Delhi 110070, India

**Keywords:** Child nutrition, India, Minimum dietary diversity, Nutrition deficiency, Nutrition policy

## Abstract

Recognising the importance of infant and young child feeding practices during the first 2 years of life, the World Health Organization's Global Nutrition Monitoring Framework developed a minimum dietary diversity (MDD) indicator for feeding children aged 6–23 months. MDD is defined as the consumption of food items from five or more groups out of a total of eight food groups. Food intake from less than five food groups is considered minimum dietary diversity failure (MDDF). Using the nationally representative National Family Health Survey (NFHS) dataset, the present study assessed the trend in MDDF between 2005–6 and 2015–16 and the factors associated with MDDF among children aged 6–23 months during 2015–16. The NFHS conducted in 2005–6 and 2015–16 covered a sample of 14 419 and 74 078 children aged 6–23 months, respectively. Overall, the MDDF reduced from 87⋅4  % (95  % confidence interval (95  % CI) 86⋅8  %, 87⋅9  %) in 2005–6 to 80⋅6  % (95  % CI 80⋅1  %, 81⋅0  %) in 2015–16. Multivariable logistic regression analysis revealed that increased child's age, second and third birth order children, higher maternal age and education, mass media exposure of mothers and more than four antenatal care visits had a negative association with the MDDF. Children living in rural areas and residing in high-focus states of India were observed with higher odds of experiencing MDDF. Exposure to community healthcare services was negatively associated with MDDF, and anaemic children were more likely to have MDDF. Socioeconomic status of mothers and children and encouragement of maternal and child healthcare use could be helpful in devising context-specific intervention to mitigate MDDF.

## Introduction

Globally, India has the highest number of under-five malnourished children^(1)^. India needs a multipronged approach to address the high prevalence of undernutrition among children^(2–4)^. Strengthening infant and young child feeding (IYCF) practices among children aged 6–23 months is crucial for reducing the burden of undernutrition and the associated morbidity and mortality^(5,6)^. To guide standard IYCF practices globally, a minimum dietary diversity (MDD) indicator was developed by experts from the World Health Organization (WHO) and the United Nations Children's Fund (UNICEF) under the ambit of the WHO Global Nutrition Monitoring Framework targets for 2025^(6)^. MDD comprises eight food groups, and in the case of food intake from five or more groups, the child is considered to be receiving the optimal level of food required for their overall development^(6)^. However, failing to receive the minimum required dietary diversity (less than five food groups) is counted as minimum dietary diversity failure (MDDF) for a child. The purpose of developing MDD indicators was three-fold: (1) compare feeding practices among regions over time; (2) identify vulnerable population groups and prioritise intervention strategies; (3) monitor progress and evaluate intervention impacts^(6,7)^.

India has been slow in adopting recommended IYCF practices^(8)^, and the performance of MDD in India has been poor for various reasons^(4,7,9,10)^. According to the Global Burden of Disease Study, India had an estimated 19⋅1 % of children (equivalent to an estimated 5 351 900 infants under 6 months of age) who were not exclusively breastfed in 2018^(11)^. Children with MDDF had low fruit, vegetable and protein-rich food consumption, and many children who met MDD also had low protein-rich food consumption^(9)^. Such statistics call for the assessment of factors that could influence MDDF. Using appropriate MeSH (Medical Subject Headings) terms, we searched PubMed bibliographic database for the recent studies in India based on nationally representative data analysing the trend, regional variation and determinants of MDDF (including eight food groups as opposed to seven food groups) among children. The search did not yield any study which covered all these aspects. The present study, thus, provides an account of the temporal change in the prevalence of MDDF among children aged 6–23 months between 2005–6 and 2015–16 and factors associated with MDDF during 2015–16 using nationally representative data. The study also presents the district-level variation in MDDF across thirty-seven states/union territories of India during 2015–16.

## Methods

### NFHS dataset

Two waves of National Family Health Survey (NFHS) datasets collected during 2005–6 (NFHS-3)^(12)^ and 2015–16 (NFHS-4)^(13)^ were used. The NFHS provides cross-sectional data and covers a nationally representative sample. The NFHS is conducted under the aegis of the Ministry of Health and Family Welfare, Government of India. The data from these surveys are widely used to inform national public health policy and design national public health interventions. By virtue of the sampling design, estimates from NFHS-3 and NFHS-4 are comparable^(14)^. NFHS-3 was designed to provide state-level estimates covering all twenty-nine states/union territories of India in 2005–6, while NFHS-4 additionally includes district-level estimates covering all thirty-seven states/union territories of India in 2015–16. NFHS-3 and NFHS-4 use a sampling frame of India's 2001 Census and 2011 Census, respectively. NFHS-3 covered 124 385 women aged 15–49 years and 69 751 men aged 15–54 years living in 109 041 households, whereas NFHS-4 sampled 699 686 women and 112 122 men of the same age group, and both surveys had over a 95 % response rate. Total 48 084 living children aged 0–59 months were covered in NFHS-3, and NFHS-4 included 244 508 children of the same age group. Further details about the NFHS sampling procedure can be found in its published reports^(12,13)^.

To fulfil the study objective, a total sample of 14 419 and 74 078 children aged 6–23 months were included in the study from NFHS-3 and NFHS-4, respectively. The trend of MDDF between 2005–6 and 2015–16 was assessed in twenty-nine states/union territories with comparable geographical areas in both NFHS-3 and NFHS-4. However, the burden of MDDF and its factors were examined for thirty-seven states/union territories covering 640 districts, using the NFHS-4 dataset. The analysis of predictors of MDDF using recent rounds of NFHS (2015–16) data helped understand the latest dynamics of MDDF and how the current programme and policy environment may help to improve the effective coverage of MDD. The prevalence of MDDF was mapped across 111 aspirational districts, 304 districts belonging to high-focus states (Assam, Bihar, Chhattisgarh, Jharkhand, Madhya Pradesh, Orissa, Rajasthan, Uttarakhand and Uttar Pradesh) and 336 districts from non-high-focus states. While performing the analysis, measures were taken to deal with sample selection bias which made the present study robust with high external validity. The Government of India identified the Aspirational Districts based on the poor performance on forty-nine key indicators, of which improvement of health and nutrition, education, agriculture and water resources, financial inclusion and skill development, and basic infrastructure are of prime concerns^(15)^. Due to the high fertility and mortality indicators, the Government of India has identified nine high-focus states which account for about 48 % of India's population^(16)^.

### Minimum dietary diversity failure

The computation of MDDF among children aged 6–23 months was guided by the definition developed by the WHO in 2008^(17)^ and its subsequent modification in 2017^(6)^. Recognising the importance of IYCF practices, which directly affect the nutritional status of children under 2 years of age, WHO's Global Nutrition Monitoring Framework developed the minimum dietary diversity (MDD) measure required for children aged 6–23 months. The WHO identifies eight diverse food groups, namely (1) breast milk, (2) grains, roots and tubers, (3) legumes and nuts, (4) dairy products, (5) flesh foods, (6) eggs, (7) vitamin A-rich fruits and vegetables and (8) other fruits and vegetables, for a standard IYCF practice. Children who receive foods from less than five of these food groups are regarded as having MDDF^(6)^.

In both NFHS-3 and NFHS-4, mothers with their youngest child (born after 2003 or later and 2014 or later, respectively) were asked about food or drink given to the child on the previous ‘day or night, either separately or combined with other foods’. Responses from mothers were used to determine if each child had at least one food from eight food groups – (1) breastmilk (one survey item: currently breastfeeding), (2) grains, roots and tubers (three survey items: fortified baby food; bread, noodles or other food made from grains; potatoes, cassava or other tubers), (3) beans, peas, lentils and nuts (one survey item), (4) dairy (three survey items: tinned, powdered or fresh milk; baby formula; cheese, yogurt or other milk products), (5) flesh foods (four survey items: chicken, duck or other birds; liver, heart or other organs; fish or shellfish; any other meat), (6) eggs (one survey item), (7) vitamin A-rich fruits or vegetables (three survey items: pumpkin, carrots and squash (yellow or orange inside); dark green leafy vegetables; ripe mangoes, papayas or other vitamin A-rich fruits) and (8) other fruits and vegetables (one survey item: any other fruits)^(7)^. A negligible proportion (<1 %) of mothers responded ‘don't know’ against the food given to their children, and those responses are considered as their children were not given that food in the present study. Such an approach does not affect the overall analysis, as documented in earlier studies^(7,18)^.

### Covariables

While the trend in MDDF (between 2005–6 and 2015–16) was analysed using NFHS-3 and NFHS-4 data, the factors associated with MDDF were examined using NFHS-4 data where a range of covariables were considered. The covariables were categorised into five groups: child characteristics (age, sex and birth order); maternal characteristics (age, education, age at firth birth, mass media exposure, number of antenatal care visits and skilled attendance at birth); household characteristics (religion, social group and wealth index); healthcare characteristics [had a health check-up from the *Anganwadi*/Integrated Child Development Services (ICDS) centre, received counselling from the A*nganwadi*/ICDS worker or auxiliary nurse midwife (ANM), received food from the *Anganwadi*/ICDS centre and whether the child had health card]; concurrent health status (fever, cough, diarrhoea and anaemia); regional characteristics (place of residence, and state of residence) among children 6–23 months. Depending on the information available in the NFHS-4 dataset, the choice of these covariables was guided by existing literature on determinants of child nutritional status.

In NFHS-4, women were asked about their frequency of exposure (not at all, less than once a week, at least once a week, almost every day) to three types of mass media (newspaper or magazine, radio, television)^(19)^. Women who responded to have exposure to any of these three media were labelled as having some mass media exposure. Whether the childbirth was attended by skilled personnel^(20)^ was considered one of the covariables, as this information indicates the maternal exposure to healthcare services that may help in adopting good IYCF practices. The construction of the wealth index was guided by the computation method developed by the Demographic and Health Survey Program^(21)^. The wealth index, a variable provided with the NFHS-4 dataset, is a composite index representing household economic status constructed using household assets and durables.

Information on whether the child had a health check-up from the *Anganwadi*/ICDS centre in the last 12 months was collected in NFHS-4. In addition, mothers were asked if they received any counselling from the *Anganwadi*/ICDS worker or ANM in the last 12 months. Also, information on the frequency of receipt of food from the *Anganwadi*/ICDS centre for children was also recorded. *Anganwadi* (synonymous with courtyard shelter) were established by the Indian government in 1975 as part of the ICDS programme to combat child hunger and malnutrition^(22)^. Under the ICDS, the *Anganwadi* platform was designed to work as the preliminary village or habitation resource for health, nutrition and early learning where *Anganwadi* Workers (AWW), assisted by helpers (AWH), look after various reproductive, maternal and child healthcare activities, including nutrition supplementation for children aged 0–6 years, and nutrition and health education for women between 15 and 49 years of age^(22)^. Whether the child had a health card was recorded in NFHS-4. Having a health card from the Department of Health and Family Welfare, the Government of India indicates that the child is enrolled with the nearest public healthcare facility and under regular observation by healthcare workers. Information on whether the child had any transitory illness (namely fever, cough and diarrhoea) in the 2 weeks preceding the survey date was asked to mothers in NFHS-4. This information was included as potential covariables because transitory illness may affect IYCF practices. NFHS-4 measured the haemoglobin (Hb) level among children aged 6–59 months, and for the present study, we used the information for children aged 6–23 months. Children with Hb of <11 g/dl were regarded as anaemic^(23)^. The protocol for measuring Hb can be furnished elsewhere^(13)^.

Selected covariables included in the analysis have ‘unknown/inconsistent response’ (e.g. number of antenatal care visits). This category was included to avoid sample exclusion, leading to sample selection bias. Although the exclusion of the sample did not yield a different result from multivariable analysis, we reported the results with a category of ‘unknown/ inconsistent response’ in selected covariables.

### Statistical analysis

Descriptive and multivariable analyses are carried out to attain the study objective. The prevalence of MDDF was estimated for eight food groups and the food items under each food group. Similarly, the state-wise and district-wise prevalence of MDDF were estimated. A multivariable binary logistic regression model was developed to understand the factors associated with MDDF among children aged 6–23 months. Variables included in the logistic regression model were checked for multicollinearity by estimating the variance inflation factor (VIF). The VIF of <5 is indicative of a low possibility of multicollinearity. Appropriate sample weighting available with the NFHS dataset were used to carry out the descriptive and multivariable analysis. The statistical software – Stata Version 14^(24)^ was used to execute the analysis. The odds ratio with *P* < 0⋅05 (two-tailed) obtained from multivariable regression was discussed in the study. The estimated prevalence of MDDF across 640 districts of India was mapped using R software, separately for aspirational districts, districts belonging to high-focus states, and districts belonging to non-high-focus states.

## Results

**Fig. 1.**
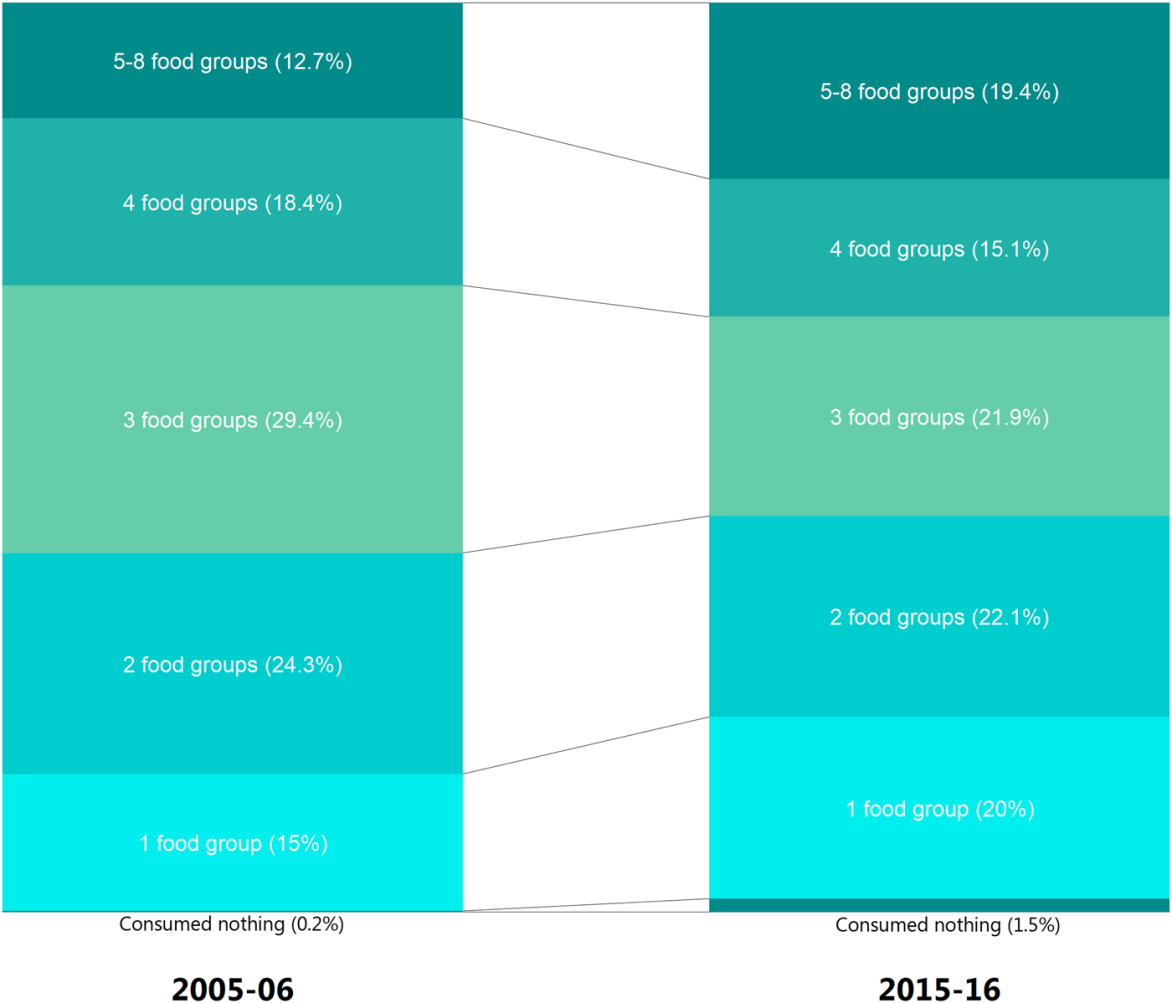
Dietary diversity (percentage distribution) among children aged 6–23 months by a number of food groups, during 2005–6 and 2015–16.

Table 1 presents the prevalence of dietary diversity and MDDF among children aged 6–23 months by eight food groups and respective underlying food items. Overall, MDDF has reduced from 87⋅4 % (95 % confidence interval (95 % CI) 86⋅8 %, 87⋅9 %) in 2005–6 to 80⋅6 % (95 % CI 80⋅1 %, 81⋅0 %) in 2015–16. However, feeding on four food groups – breastfeeding; grains, roots and tubers; legumes and nuts; and dairy products – reduced, whereas an increase was observed in the feeding of flesh foods, eggs, Vitamin A-rich fruits and vegetables, and other fruits and vegetables. Between 2005–6 and 2015–16, feeding of children of one food group and five or more food groups increased by 5 and 6 percentage points, respectively (Fig. 1). Consequently, the distribution of children fed on two through four food groups declined.

**Table 1.** Prevalence (%) with 95 % confidence interval (CI) of dietary diversity and MDDF among children aged 6–23 months by eight food groups and the underlying food items, India, 2005–6 and 2015–16

	2005–6, % (95 % CI)	2015–16, % (95 % CI)
Breastmilk	**87⋅1** (**86⋅5**–**87⋅6)**	**84⋅4** (**84⋅2**–**84⋅7)**
Currently breastfeeding	87⋅1 (86⋅5–87⋅6)	84⋅4 (84⋅2–84⋅7)
Grains, roots and tubers	**78⋅5** (**77⋅9–79⋅2)**	**68⋅7** (**68⋅4–69⋅1)**
Any commercially fortified baby food such as Cerelac or Farex	15⋅5 (15⋅0–16⋅1)	15⋅6 (15⋅4–15⋅9)
Any bread, *roti*, *chapati*, rice, noodles, biscuits, *idli* or any other foods made from grains	71⋅4 (70⋅7–72⋅1)	62⋅9 (62⋅5–63⋅2)
Any white potatoes, white yams, cassava or any other foods made from roots	21⋅9 (21⋅3–22⋅6)	21⋅3 (21⋅0–21⋅6)
Legumes and nuts	**13⋅8** (**13⋅2–14⋅3)**	**13⋅3** (**13⋅0–13⋅5)**
Any foods made from beans, peas, lentils and nuts	13⋅8 (13⋅2–14⋅3)	13⋅3 (13⋅0–13⋅5)
Dairy products	**54⋅0** (**53⋅2–54⋅8)**	**50⋅6** (**50⋅2–51⋅0)**
Any other milk such as tinned, powdered or fresh animal milk	47⋅4 (46⋅7–48⋅2)	40⋅1 (39⋅7–40⋅4)
Commercially produced infant formula	10⋅2 (9⋅7–10⋅6)	10⋅4 (10⋅2–10⋅6)
Any cheese, yoghurt or other milk products^a^	10⋅9 (10⋅4–11⋅4)	16⋅5 (16⋅3–16⋅8)
Flesh foods	**7⋅5** (**7⋅1–7⋅9)**	**10⋅0** (**9⋅7–10⋅2)**
Any chicken, duck or other birds	1⋅2 (1⋅0–1⋅4)	4⋅8 (4⋅6–4⋅9)
Any liver, kidney, heart or other organ meat	1⋅5 (1⋅3–1⋅7)	5⋅1 (5⋅0–5⋅3)
Any fresh or dried fish or shellfish	4⋅6 (4⋅2–4⋅9)	4⋅7 (4⋅6–4⋅9)
Any other meat	1⋅8 (1⋅6–2⋅0)	3⋅9 (3⋅7–4⋅0)
Eggs	**5⋅1** (**4⋅8–5⋅5)**	**14⋅4** (**14⋅1–14⋅6)**
Any egg	5⋅1 (4⋅8–5⋅5)	14⋅4 (14⋅1–14⋅6)
Vitamin A-rich fruits or vegetables	**33⋅3** (**32⋅6–34⋅1)**	**39⋅4** (**39⋅0–39⋅8)**
Any pumpkin, carrots or sweet potatoes that are yellow or orange inside	13⋅1 (12⋅6–13⋅7)	19⋅7 (19⋅4–20⋅0)
Any dark green, leafy vegetable	21⋅4 (20⋅7–22⋅0)	28⋅4 (28⋅1–28⋅8)
Any ripe mangoes, papayas, cantaloupe or jackfruit	9⋅8 (9⋅3–10⋅2)	18⋅4 (18⋅2–18⋅7)
Other fruits and vegetables	**14⋅9** (**14⋅4–15⋅5)**	**23⋅8** (**23⋅5–24⋅1)**
Any other fruits or vegetables	14⋅9 (14⋅4–15⋅5)	23⋅8 (23⋅5–24⋅1)
MDDF^b^	**87⋅4** (**86⋅8–87⋅9)**	**80⋅6** (**80⋅1–81⋅0)**

a Unlike NFHS 2005–6, NFHS 2015–16 collected information on yoghurt intake separately.

b MDDF is defined as the consumption of food from less than five food groups.

Estimates for eight major food groups and MDDF are shown in bold values.

**Fig. 2.**
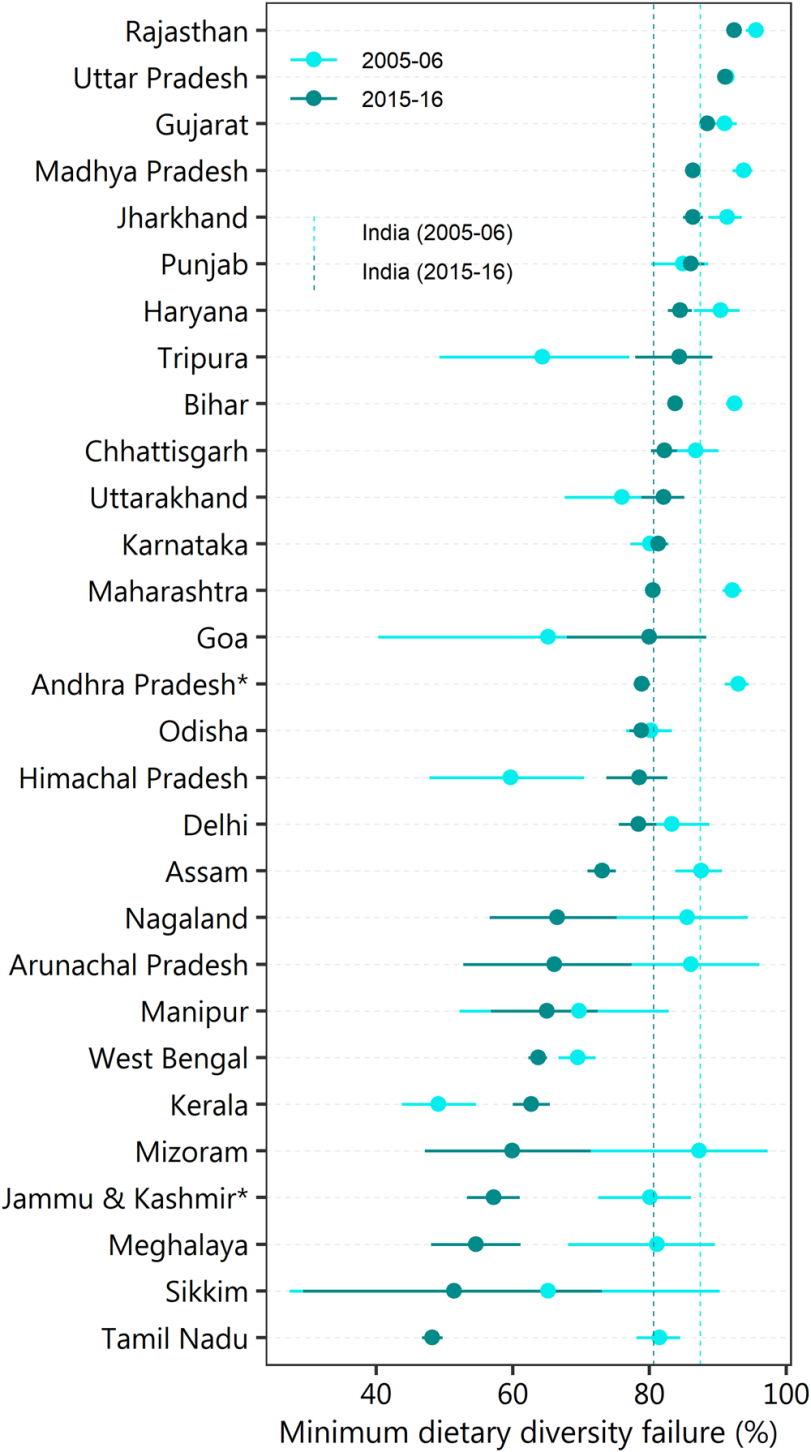
Change in the prevalence (%) of MDDF among children aged 6–23 months across major states/union territories in India between 2005–6 and 2015–16. *Estimates for Andhra Pradesh and Jammu & Kashmir include the Telangana and Ladakh union territories, respectively.

**Fig. 3.**
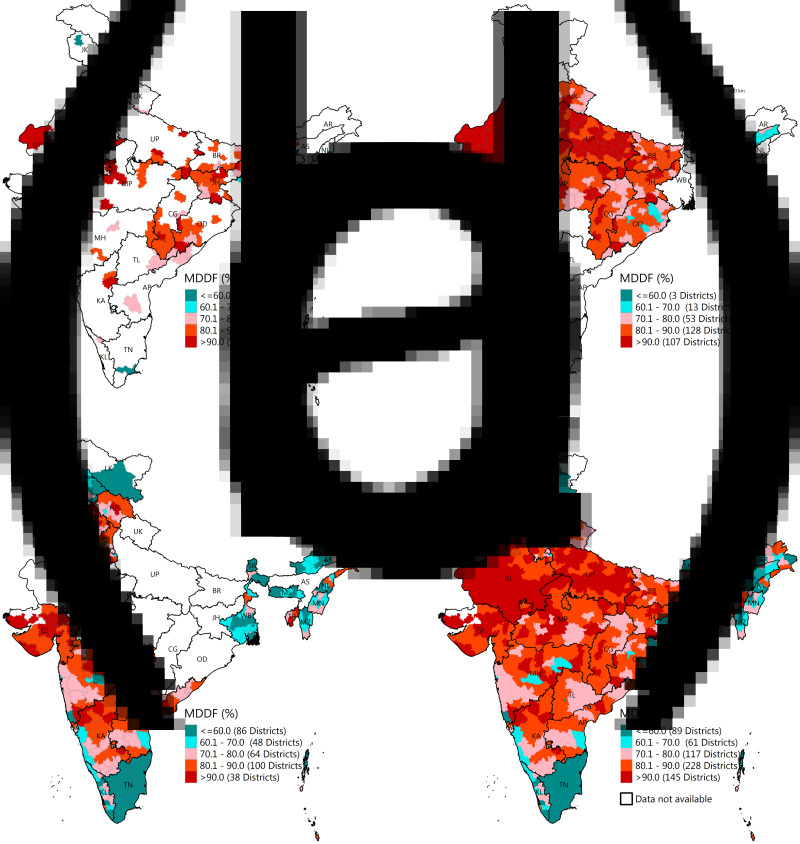
MDDF among children aged 6–23 months across districts of India, 2015–16. (a) MDDF (%) across 111 aspirational districts; (b) MDDF (%) across districts of nine high-focus group states; (c) MDDF (%) across districts of 28 non-high-focus group states/union territories and (d) MDDF (%) across 640 districts of India. AP: Andhra Pradesh, AR: Arunachal Pradesh, AS: Assam, BR: Bihar, CH: Chhattisgarh, GJ: Gujarat, HP: Himachal Pradesh, HR: Haryana, JH: Jharkhand, JK: Jammu & Kashmir, KA: Karnataka, KL: Kerala, LK: Ladakh, MH: Maharashtra, ML: Meghalaya, MN: Manipur, MP: Madhya Pradesh, MZ: Mizoram, NL: Nagaland, OD: Odisha, PB: Punjab, RJ: Rajasthan, SK: Sikkim, TL: Telangana, TN: Tamil Nadu, TR: Tripura, UK: Uttarakhand, UP: Uttar Pradesh, WB: West Bengal

Change in the prevalence of MDDF across states/union territories between 2005–6 and 2015–16 has been presented in Fig. 2 and Supplementary Table S1. The state of Rajasthan recorded the highest MDDF in both 2005–6 (95⋅6 %) and 2015–16 (92⋅4 %). The state of Tamil Nadu (33⋅3 percentage points) recorded the highest reduction in MDDF, while the lowest was observed in Uttar Pradesh (0⋅2 percentage points). Seven states (Punjab and Karnataka – 1⋅2 percentage points, Uttarakhand – 6⋅1 percentage points, Kerala – 13⋅6 percentage points, Goa – 14⋅8 percentage points, Himachal Pradesh – 18⋅8 percentage points and Tripura – 20⋅1 percentage points) accounted for an increase in MDDF. The district-level prevalence of MDDF in 2015–16 is presented in Fig. 3 with separate presentations across aspirational districts, high-focus states and non-high-focus states. Estimates for all 640 districts are available in Supplementary Table S2. The lowest prevalence of MDDF was recorded in the South Garo Hills district of Meghalaya state (21⋅6 %), while the highest prevalence was recorded in the union territory of Dadra and Nagar Haveli (100 %). Total twenty-three districts of all aspirational districts, 107 in high-focus states and thirty-eight in non-high-focus states had over 90 % of children with MDDF.

Table 2 presents the prevalence of MDDF by selected background characteristics along with the effect measures (adjusted odds ratios) based on the multivariable logistic regression model. As high as 90⋅3 % (95 % CI 89⋅7 %, 90⋅8 %) of children aged 6–11 months and 85⋅1 % (95 % CI 84⋅4 %, 85⋅8 %) of children of mothers with no or incomplete primary education had MDDF. Children living in rural areas had higher MDDF as compared to their urban counterparts. Anaemic children were found to have a higher prevalence of MDDF (81⋅4 %, 95 % CI 80⋅9 %, 82⋅0 %) than non-anaemic (77⋅3 %, 95 % CI 76⋅4, 78⋅2). Adjusted odds ratio (aOR) estimated using the binary logistic regression model revealed the factors associated with MDDF (Table 2). The estimated VIF of <5 indicated a low probability of multicollinearity among variables in the regression model. A lower likelihood for MDDF was observed among children aged 12–17 months (aOR: 0⋅35, 95 % CI 0⋅33, 0⋅39, *P* < 0⋅001) and children aged 18–23 months (aOR: 0⋅28, 95 % CI 0⋅26, 0⋅30, *P* < 0⋅001) compared to children aged 6–11 months. Children of the first birth order had higher odds of having MDDF than the children of the second and third birth order. With the increase in maternal age and maternal education, children were less likely to experience MDDF, whereas the children of mothers with some exposure to mass media (aOR: 0⋅84, 95 % CI 0⋅78, 0⋅91, *P* < 0⋅001) and with four or more antenatal care (ANC) visits during their pregnancy (aOR: 0⋅86, 95 % CI 0⋅80, 0⋅92, *P* < 0⋅001) had a protective association with MDDF. Children from Hindu families were more likely to have MDDF than those from Muslim and Christian families. Similarly, children who belonged to ‘others’ (non-reserved) social groups had higher odds of being MDDF than those belonging to scheduled castes, scheduled tribes and other backward classes. An increased odds of MDDF was observed among children who lived in rural areas (aOR: 1⋅20, 95 % CI 1⋅11, 1⋅30, *P* < 0⋅001) and those who were residing in high-focus states (aOR: 1⋅92, 95 % CI 1⋅80, 2⋅05, *P* < 0⋅001) than their counterparts. The prevalence of MDDF was marginally higher among children from the poorest economic group; however, when accounting for all potential predictors, they were less likely to have MDDF than children from the poorer, middle, richer and richest economic groups. Healthcare exposure among mothers (health check-up from the *Anganwadi*/ICDS centre, counselling from the *Anganwadi*/ICDS worker or ANM and having health cards) had a protective impact on children against MDDF. Anaemic children were more likely to experience MDDF (aOR: 1⋅20, 95 % CI 1⋅13, 1⋅28, *P* < 0⋅001) than non-anaemic children.

**Table 2.** Prevalence (%) of and adjusted odds ratios (aOR) for MDDF among children aged 6–23 months by the child, maternal, household, health exposure, health status and regional characteristics, India, 2015–16

	*n*	% (95 % CI)	aOR (95 % CI)	*P-*value
Child characteristics				
Age (in months)				
6–11	25 199	90⋅3 (89⋅7–90⋅8)	1⋅00 (referent)	
12–17	24 701	77⋅2 (76⋅3–78⋅1)	0⋅35 (0⋅33–0⋅39)	<0⋅001
18–23	24 178	73⋅8 (72⋅9–74⋅7)	0⋅28 (0⋅26–0⋅30)	<0⋅001
Sex				
Male	38 717	80⋅7 (80⋅1–81⋅3)	1⋅00 (referent)	
Female	35 361	80⋅3 (79⋅6–81⋅0)	0⋅99 (0⋅93–1⋅05)	0⋅755
Birth order				
1	27 420	80⋅7 (79⋅9–81⋅4)	1⋅00 (referent)	
2	23 589	78⋅1 (77⋅2–79⋅0)	0⋅85 (0⋅79–0⋅91)	<0⋅001
3	11 806	81⋅0 (79⋅9–82⋅1)	0⋅88 (0⋅80–0⋅98)	0⋅015
≥4	11 263	85⋅7 (84⋅7–86⋅6)	1⋅05 (0⋅93–1⋅19)	0⋅423
Maternal characteristics				
Age (in completed years)				
15–24	31 643	81⋅8 (81⋅1–82⋅5)	1⋅00 (referent)	
25–34	37 350	79⋅3 (78⋅6–80⋅0)	0⋅88 (0⋅82–0⋅94)	<0⋅001
35–49	5085	80⋅9 (78⋅4–83⋅2)	0⋅81 (0⋅68–0⋅97)	0⋅023
Education				
No or incomplete primary	25 466	85⋅1 (84⋅4–85⋅8)	1⋅00 (referent)	
Primary or incomplete secondary	34 080	79⋅8 (79⋅1–80⋅4)	0⋅84 (0⋅78–0⋅92)	<0⋅001
Secondary or higher	14 532	75⋅1 (73⋅8–76⋅2)	0⋅62 (0⋅55–0⋅69)	<0⋅001
Age at first birth (in completed years)				
<18	7905	80⋅7 (79⋅4–82⋅0)	1⋅00 (referent)	
≥18	66 173	80⋅5 (80⋅0–81⋅0)	1⋅03 (0⋅93–1⋅14)	0⋅543
Mass media exposure				
None	20 043	86⋅4 (85⋅7–87⋅0)	1⋅00 (referent)	
Some	54 035	78⋅5 (77⋅9–79⋅1)	0⋅84 (0⋅78–0⋅91)	<0⋅001
Number of ANC visit				
<4	37 198	84⋅6 (84⋅1–85⋅1)	1⋅00 (referent)	
≥4	33 332	75⋅9 (75⋅1–76⋅6)	0⋅86 (0⋅80–0⋅92)	<0⋅001
Unknown/inconsistent response	3548	88⋅8 (87⋅1–90⋅3)	ns	
Skilled attendance at birth				
Safe	58 464	79⋅8 (79⋅2–80⋅3)	1⋅00 (referent)	
Unsafe	15 614	84⋅1 (83⋅2–85⋅0)	0⋅96 (0⋅89–1⋅05)	0⋅359
Household characteristics				
Religion				
Hinduism	53 553	81⋅3 (80⋅8–81⋅8)	1⋅00 (referent)	
Islam	11 665	78⋅3 (77⋅1–79⋅4)	0⋅81 (0⋅74–0⋅89)	<0⋅001
Christianity	6052	69⋅5 (66⋅1–72⋅7)	0⋅70 (0⋅58–0⋅84)	<0⋅001
Others	2808	80⋅1 (76⋅7–83⋅0)	1⋅20 (0⋅96–1⋅49)	0⋅103
Social group				
Others	13 278	80⋅3 (79⋅0–81⋅4)	1⋅00 (referent)	
Scheduled castes	14 160	80⋅8 (79⋅8–81⋅9)	0⋅86 (0⋅77–0⋅96)	0⋅005
Scheduled tribes	14 692	81⋅1 (80⋅0–82⋅3)	0⋅83 (0⋅73–0⋅94)	0⋅003
Other backward classes	29 123	81⋅4 (80⋅8–82⋅0)	0⋅89 (0⋅81–0⋅97)	0⋅01
Unknown/inconsistent response	2825	68⋅1 (65⋅0–71⋅2)	ns	
Wealth index				
Poorest	18 814	85⋅0 (84⋅3–85⋅7)	1⋅00 (referent)	
Poorer	17 203	82⋅0 (81⋅1–82⋅9)	1⋅11 (1⋅01–1⋅21)	0⋅028
Middle	15 061	79⋅9 (78⋅9–80⋅9)	1⋅22 (1⋅10–1⋅35)	<0⋅001
Richer	12 642	76⋅2 (74⋅8–77⋅5)	1⋅16 (1⋅03–1⋅31)	0⋅015
Richest	10 358	77⋅4 (75⋅9–78⋅8)	1⋅39 (1⋅21–1⋅59)	<0⋅001
Healthcare exposure				
Had a health check-up from the *Anganwadi*/ICDS centre				
No	41 345	83⋅0 (82⋅4–83⋅6)	1⋅00 (referent)	
Yes	32 271	77⋅6 (76⋅9–78⋅3)	0⋅84 (0⋅77–0⋅93)	0⋅001
Unknown/inconsistent response	462	83⋅5 (78⋅5–87⋅6)	ns	
Received counselling from the *Anganwadi*/ICDS worker or ANM				
No	51 958	82⋅5 (82⋅0–83⋅0)	1⋅00 (referent)	
Yes	21 483	76⋅1 (75⋅2–77⋅0)	0⋅85 (0⋅79–0⋅93)	<0⋅001
Unknown/inconsistent response	637	83⋅4 (79⋅3–86⋅8)	ns	
Received food from the *Anganwadi*/ICDS centre				
Not at all	32 478	82⋅7 (81⋅9–83⋅4)	1⋅00 (referent)	
Almost daily	12 278	74⋅6 (73⋅4–75⋅7)	0⋅94 (0⋅83–1⋅06)	0⋅324
At least once a week	11 279	81⋅3 (80⋅3–82⋅4)	1⋅11 (0⋅98–1⋅25)	0⋅087
At least once a month	14 711	80⋅8 (79⋅8–81⋅7)	1⋅09 (0⋅98–1⋅22)	0⋅110
Less often	3219	83⋅4 (81⋅5–85⋅1)	1⋅15 (0⋅99–1⋅34)	0⋅077
Unknown/inconsistent response	113	81⋅9 (70⋅6–89⋅5)	ns	
Have health card				
No	5746	87⋅1 (85⋅6–88⋅5)	1⋅00 (referent)	
Yes	62 593	79⋅7 (79⋅2–80⋅2)	0⋅81 (0⋅70–0⋅93)	0⋅004
Not required	5739	84⋅3 (83⋅0–85⋅6)	0⋅96 (0⋅80–1⋅14)	0⋅608
Concurrent health status				
Had fever				
No	61 299	80⋅7 (80⋅2–81⋅2)	1⋅00 (referent)	
Yes	12 733	79⋅8 (78⋅7–80⋅8)	1⋅01 (0⋅92–1⋅10)	0⋅873
Unknown/inconsistent response	46	73⋅6 (52⋅3–87⋅6)	ns	
Had cough				
No	63 336	81⋅0 (80⋅5–81⋅5)	1⋅00 (referent)	
Yes	10 709	77⋅5 (76⋅3–78⋅7)	0⋅76 (0⋅69–0⋅83)	<0⋅001
Unknown/inconsistent response	33	99⋅4 (97⋅9–99⋅8)	ns	
Had diarrhoea				
No	63 540	80⋅4 (79⋅9–80⋅9)	1⋅00 (referent)	
Yes	10 476	81⋅2 (80⋅1–82⋅4)	0⋅93 (0⋅85–1⋅02)	0⋅119
Unknown/inconsistent response	62	80⋅2 (63⋅8–90⋅3)	ns	
Anaemic				
No	21 304	77⋅3 (76⋅4–78⋅2)	1⋅00 (referent)	
Yes	47 371	81⋅4 (80⋅9–82⋅0)	1⋅20 (1⋅13–1⋅28)	<0⋅001
Unknown/inconsistent response	5403	84⋅5 (82⋅4–86⋅4)	ns	
Regional characteristics				
Place of residence				
Urban	17 578	76⋅1 (74⋅9–77⋅3)	1⋅00 (referent)	
Rural	56 500	82⋅3 (81⋅8–82⋅7)	1⋅20 (1⋅11–1⋅30)	<0⋅001
State of residence				
Non-high-focus states	29 624	73⋅3 (72⋅4–74⋅2)	1⋅00 (referent)	
High-focus states	44 454	86⋅7 (86⋅3–87⋅0)	1⋅92 (1⋅80–2⋅05)	<0⋅001
Total	74 078	80⋅6 (80⋅1–81⋅0)		

ANC, antenatal care; ANM, auxiliary nurse midwife; CI, confidence interval; ICDS, Integrated Child Development Services; *n*, sample size; ns, not shown; *P*-value, level of significance.

## Discussion

Using a nationally representative cross-sectional dataset from India, the present study assessed the MDDF among children aged 6–23 months and its associated factors. Findings revealed that nearly four in every five children aged 6–23 months in India experienced MDDF in 2015–16, recording a relatively small improvement (a decline of seven percentage points) between 2005–6 and 2015–2016. With developed states such as Kerala, Goa and Karnataka reporting an increase in MDDF between 2005–6 and 2015–16, as well as large clusters of districts with high MDDF covering highly populated states in India, poor IYCF practices among children in India seem widespread. Recent studies^(25,26)^ also identified that strengthening IYCF programmes would require intensive focused intervention to control MDDF in select districts, especially aspirational districts in high-focus group states in India. The present study presents the association of MDDF with a range of factors, including child, maternal, household and regional characteristics of children, their healthcare exposure and concurrent health status.

With increasing age, children were less likely to experience MDDF. This association is consistent with previous studies conducted in India^(27)^ and in the Amhara region, Addis Zemen town, Ethiopia^(28)^. The propensity of MDDF among older children is low primarily for two reasons. First, the frequency of food consumption among older children is relatively higher, which helps diversify food groups^(28)^. Second, the quantity of food requirement among younger children compared to older is low, decreasing the chance of food diversification among young children^(29)^. Children of the first birth order had higher odds of experiencing MDDF than the children of the second and third birth order. The role of birth order in feeding practices in India has been documented in an earlier study^(27)^, highlighting that mothers or caretakers with a child of the first birth order tend to be inexperienced about appropriate feeding practices.

The children experiencing MDDF were negatively associated with their mother's age and education. The increasing age of mothers portends their adequate experience in rearing children and being aware of their feeding practices, suggestive of reducing the prevalence of MDDF among their children. The effect of education on child nutrition has been widely studied^(30)^. Maternal education is directly linked with children's appropriate health and awareness about child nutrition^(31)^. Similarly, mothers’ mass media exposure was also found to reduce MDDF, consistent with the findings of other studies on MDD in India^(27,32)^.

Children from Hindu families were more likely to experience MDDF than non-Hindu (Muslim and Christian) families. Results also revealed that children from privileged social groups (non-scheduled or backward class) and better economic groups were more likely to experience MDDF. These findings have also been reported in other studies conducted in India^(27)^. However, there is no explanation available in existing literature and calls for further investigation. Existing literature on the poor nutrition outcomes of children in higher socioeconomic groups, particularly related to WASH and sanitation issues, may echo our findings on poor awareness and adoption of health-promoting practices^(33)^. Children living in rural areas and residing in high-focus group states were more likely to have MDDF. A regional analysis^(34)^ showed how MDD could vary by region in India. A similar explanation applies to children from high-focus group states^(16)^ who are more likely to be nutritionally compromised.

Multivariable analysis also revealed that healthcare exposure could influence the reduction of MDDF. Women who had four or more ANC visits during pregnancy witnessed lower odds of MDDF among their children, as observed in an earlier study^(34)^. Four or more ANC visits are indicative of maternal access to required healthcare and healthcare information on child nutrition. Multivariable results also indicate that the health check-up of children from the *Anganwadi*/ICDS centre, counselling of mothers from the *Anganwadi*/ICDS worker or ANM and having a health card for children, all had a negative association with MDDF. Access to these services is indicative of access to appropriate maternal and child healthcare services^(27,31,34)^. Children having coughs in the recent past had lower odds of MDDF. Giving children a diverse group of foods to overcome the illness could be a possible reason for lower MDDF. Findings also suggest that anaemic children are more likely to experience MDDF. This relationship could be a reverse causation as well. However, anaemic people often have less appetite for food^(35)^, which might explain the higher MDDF among anaemic children.

The findings of the present study should be understood considering a few possible limitations. First, due to the unavailability of information with the NFHS dataset, the present study could not include all the potential factors associated with MDDF. For example, information on the accessibility and availability of food groups would have clarified the poor coverage of MDDF. Also, information on the breastfeeding pattern of children would have been more helpful in understanding the overall coverage of MDD. Second, since most of the information is based on mothers’ recall, this information could be affected by recall errors or social desirability bias. Third, the MDDF is based on 24-h recall, which might be influenced by factors such as if the child ate out with parents the previous night. Despite these limitations, the present study is the first of its kind to use the revised guideline for MDDF with eight food groups and using nationally representative data, which provided robust estimates with high external validity. To conclude, India has an unacceptably high prevalence of MDDF among children, potentially hampering the overall development of child health in India. To reduce the burden of MDDF, although challenging, a context-specific intervention for taking-up MDD is imperative.

## Supplementary material

For supplementary material accompanying this paper visit https://doi.org/10.1017/jns.2022.2. click here to view supplementary material
