# Supplementary material for: Factors associated with minimum dietary diversity failure among Indian children
Source: J Nutr Sci. 2022 Feb 4;11:e4. doi: 10.1017/jns.2022.2 (PMC8889227; doi:10.1017/jns.2022.2)
Supplement: Supplementary file 1 [file S2048679022000027sup001.docx]

**Factors associated with minimum dietary diversity failure among Indian children**

Rajesh Kumar Rai, Sandhya S Kumar, Chandan Kumar

**Supplementary material**

| **Supplementary Table 1**. Burden of minimum dietary diversity failure (MDDF) among children aged 6-23 months in India, during 2005-06 and 2015-16. | | | |
| --- | --- | --- | --- |
| **States/ union territories** | **MDDF in 2005-06,**  **% (95% CI)** |  | **MDDF in 2015-16,**  **% (95% CI)** |
| Andhra Pradesh (including Telangana) ^¥^ | 93.0 (91.0-94.5) |  | 78.9 (77.7-80.1) |
| Arunachal Pradesh | 86.1 (60.8-96.1) |  | 66.1 (52.7-77.4) |
| Assam | 87.6 (83.8-90.6) |  | 73.1 (70.9-75.1) |
| Bihar | 92.5 (91.2-93.6) |  | 83.8 (83.0-84.5) |
| Chhattisgarh | 86.8 (82.5-90.1) |  | 82.2 (80.2-84.0) |
| Delhi | 83.3 (75.9-88.8) |  | 78.4 (75.5-81.0) |
| Goa | 65.2 (40.3-83.9) |  | 80.0 (67.9-88.3) |
| Gujarat | 91.0 (88.6-92.8) |  | 88.5 (87.3-89.6) |
| Haryana | 90.4 (86.5-93.2) |  | 84.5 (82.7-86.2) |
| Himachal Pradesh | 59.7 (47.8-70.4) |  | 78.5 (73.7-82.6) |
| Jammu & Kashmir (including Ladakh) ^±^ | 80.1 (72.5-86.1) |  | 57.2 (53.3-61.0) |
| Jharkhand | 91.4 (88.6-93.5) |  | 86.4 (84.9-87.8) |
| Karnataka | 80.1 (77.2-82.8) |  | 81.3 (79.9-82.6) |
| Kerala | 49.1 (43.7-54.6) |  | 62.7 (60.0-65.4) |
| Madhya Pradesh | 93.8 (92.2-95.1) |  | 86.4 (85.4-87.3) |
| Maharashtra | 92.2 (90.7-93.5) |  | 80.5 (79.4-81.4) |
| Manipur | 69.7 (52.2-82.8) |  | 65.0 (56.8-72.4) |
| Meghalaya | 81.1 (68.1-89.6) |  | 54.6 (48.0-61.1) |
| Mizoram | 87.3 (56.8-97.3) |  | 59.9 (47.1-71.4) |
| Nagaland | 85.5 (67.4-94.4) |  | 66.5 (56.6-75.2) |
| Odisha | 80.2 (76.6-83.3) |  | 78.8 (77.1-80.4) |
| Punjab | 84.9 (80.3-88.6) |  | 86.1 (84.0-88.0) |
| Rajasthan | 95.6 (94.1-96.7) |  | 92.4 (91.6-93.2) |
| Sikkim | 65.2 (27.3-90.3) |  | 51.4 (29.3-73.0) |
| Tamil Nadu | 81.5 (78.1-84.5) |  | 48.2 (46.7-49.7) |
| Tripura | 64.3 (49.2-77.1) |  | 84.4 (77.9-89.2) |
| Uttar Pradesh | 91.3 (90.2-92.3) |  | 91.1 (90.6-91.6) |
| Uttarakhand | 76.0 (67.6-82.7) |  | 82.1 (78.8-85.1) |
| West Bengal | 69.5 (66.7-72.1) |  | 63.7 (62.3-65.0) |
|  |  |  |  |
| Andaman & Nicobar Island | nc |  | 69.7 (43.3-87.4) |
| Chandigarh | nc |  | 94.2 (81.1-98.4) |
| Dadra & Nagar Haveli | nc |  | 100.0 |
| Daman & Diu | nc |  | 79.9 (40.9-95.8) |
| Lakshadweep | nc |  | 72.7 (14.7-97.6) |
| Puducherry | nc |  | 46.7 (34.3-59.6) |
|  |  |  |  |
| Andhra Pradesh^¥^ | nr |  | 80.5 (78.9-82.0) |
| Telangana^¥^ | nr |  | 76.9 (75.0-78.6) |
| Jammu & Kashmir^±^ | nr |  | 57.2 (53.3-61.0) |
| Ladakh^±^ | nr |  | 58.4 (28.7-83.0) |
| ^¥^During NFHS 2005-06, separate estimates for Andhra Pradesh and Telangana were not available, whereas during NFHS 2015-16, separate data were collected for Andhra Pradesh and Telangana.  ^±^ Ladakh was integral part of Jammu & Kashmir during NFHS 2005-06, whereas NFHS 2015-16 collected district level information which made helped generate separate estimate for Ladakh and Jammu & Kashmir. The Union Territory Ladakh was established in 2019.  CI: confidence interval, nc: not collected; nr: not required | | | |

| **Supplementary Table 2.** Burden of minimum dietary diversity failure (MDDF) among children aged 6-23 months across 640 districts of 37 states/ union territories in India, 2015-16. | | |
| --- | --- | --- |
| **State/ union territory** | **District** | **MDDF, %** |
| **Andaman & Nicobar Island** | Nicobars | 80.0 |
|  | North & Middle Andaman | 57.6 |
|  | South Andaman | 73.6 |
|  |  |  |
| **Andhra Pradesh** | Srikakulam | 87.9 |
|  | Vizianagaram | 75.1 |
|  | Visakhapatnam | 75.4 |
|  | East Godavari | 82.2 |
|  | West Godavari | 91.2 |
|  | Krishna | 82.8 |
|  | Guntur | 79.2 |
|  | Prakasam | 65.4 |
|  | Nellore | 85.4 |
|  | Kadapa / Y. S. R. | 76.6 |
|  | Kurnool | 85.7 |
|  | Anantapur | 72.1 |
|  | Chittoor | 88.9 |
|  |  |  |
| **Arunachal Pradesh** | Tawang | 59.2 |
|  | West Kameng | 43.5 |
|  | East Kameng | 67.7 |
|  | Papumpare | 60.9 |
|  | Upper Subansiri | 49.8 |
|  | West Siang | 62.4 |
|  | East Siang | 71.8 |
|  | Upper Siang | 76.3 |
|  | Changlang | 84.6 |
|  | Tirap | 83.7 |
|  | Lower Subansiri | 52.0 |
|  | Kurung Kumey | 66.9 |
|  | Dibang Valley | 83.4 |
|  | Lower Dibang Valley | 65.2 |
|  | Lohit | 72.5 |
|  | Anjaw | 51.7 |
|  |  |  |
| **Assam** | Kokrajhar | 65.0 |
|  | Dhubri | 79.5 |
|  | Goalpara | 67.4 |
|  | Barpeta | 69.7 |
|  | Morigaon | 82.4 |
|  | Nagaon | 72.5 |
|  | Sonitpur | 75.9 |
|  | Lakhimpur | 68.3 |
|  | Dhemaji | 72.6 |
|  | Tinsukia | 67.2 |
|  | Dibrugarh | 64.3 |
|  | Sivasagar | 64.0 |
|  | Jorhat | 68.9 |
|  | Golaghat | 51.8 |
|  | Karbi Anglong | 62.8 |
|  | Dima Hasao | 80.9 |
|  | Cachar | 95.0 |
|  | Karimganj | 77.7 |
|  | Hailakandi | 76.4 |
|  | Bongaigaon | 70.8 |
|  | Chirang | 85.3 |
|  | Kamrup | 78.6 |
|  | Kamrup Metropolitan | 51.3 |
|  | Nalbari | 86.8 |
|  | Baksa | 58.9 |
|  | Darrang | 85.3 |
|  | Udalguri | 78.3 |
|  |  |  |
| **Bihar** | Pashchim Champaran | 77.7 |
|  | Purba Champaran | 85.5 |
|  | Sheohar | 82.2 |
|  | Sitamarhi | 84.2 |
|  | Madhubani | 79.3 |
|  | Supaul | 83.8 |
|  | Araria | 76.6 |
|  | Kishanganj | 78.3 |
|  | Purnia | 82.2 |
|  | Katihar | 87.5 |
|  | Madhepura | 92.0 |
|  | Saharsa | 86.5 |
|  | Darbhanga | 82.7 |
|  | Muzaffarpur | 84.7 |
|  | Gopalganj | 78.9 |
|  | Siwan | 84.4 |
|  | Saran | 77.0 |
|  | Vaishali | 83.4 |
|  | Samastipur | 86.7 |
|  | Begusarai | 86.0 |
|  | Khagaria | 84.7 |
|  | Bhagalpur | 82.2 |
|  | Banka | 86.0 |
|  | Munger | 83.4 |
|  | Lakhisarai | 86.5 |
|  | Sheikhpura | 74.5 |
|  | Nalanda | 85.3 |
|  | Patna | 90.1 |
|  | Bhojpur | 80.9 |
|  | Buxar | 80.5 |
|  | Kaimur (Bhabua) | 90.6 |
|  | Rohtas | 95.8 |
|  | Aurangabad | 83.9 |
|  | Gaya | 84.9 |
|  | Nawada | 76.3 |
|  | Jamui | 86.2 |
|  | Jehanabad | 74.6 |
|  | Arwal | 91.7 |
|  |  |  |
| **Chandigarh** | Chandigarh | 94.2 |
|  |  |  |
| **Chhattisgarh** | Korea (Koriya) | 71.9 |
|  | Surguja | 75.2 |
|  | Jashpur | 80.1 |
|  | Raigarh | 75.7 |
|  | Korba | 87.8 |
|  | Janjgir - Champa | 74.3 |
|  | Bilaspur | 82.2 |
|  | Kabirdham | 80.9 |
|  | Rajnandgaon | 81.6 |
|  | Durg | 90.1 |
|  | Raipur | 84.3 |
|  | Mahasamund | 74.0 |
|  | Dhamtari | 88.1 |
|  | Uttar Bastar Kanker | 87.1 |
|  | Bastar | 86.5 |
|  | Narayanpur | 95.3 |
|  | Dakshin Bastar Dantewada | 89.7 |
|  | Bijapur | 88.8 |
|  |  |  |
| **Dadra & Nagar Haveli** | Dadra & Nagar Haveli | 100.0 |
|  |  |  |
| **Daman & Diu** | Diu | 82.0 |
|  | Daman | 79.2 |
|  |  |  |
| **Goa** | North Goa | 84.4 |
|  | South Goa | 74.4 |
|  |  |  |
| **Gujarat** | Kachchh | 90.9 |
|  | Banaskantha | 85.0 |
|  | Patan | 93.6 |
|  | Mahesana | 90.8 |
|  | Sabarkantha | 92.0 |
|  | Gandhinagar | 93.1 |
|  | Ahmadabad | 90.3 |
|  | Surendranagar | 96.2 |
|  | Rajkot | 88.9 |
|  | Jamnagar | 80.1 |
|  | Porbandar | 94.2 |
|  | Junagadh | 93.2 |
|  | Amreli | 84.6 |
|  | Bhavnagar | 89.2 |
|  | Anand | 90.1 |
|  | Kheda | 87.0 |
|  | Panchmahal | 91.3 |
|  | Dohad | 86.9 |
|  | Vadodara | 84.6 |
|  | Narmada | 94.5 |
|  | Bharuch | 84.9 |
|  | The Dangs | 89.3 |
|  | Navsari | 86.9 |
|  | Valsad | 82.9 |
|  | Surat | 85.0 |
|  | Tapi | 86.6 |
|  |  |  |
| **Haryana** | Panchkula | 87.9 |
|  | Ambala | 80.2 |
|  | Yamunanagar | 81.4 |
|  | Kurukshetra | 85.1 |
|  | Kaithal | 93.9 |
|  | Karnal | 85.5 |
|  | Panipat | 84.4 |
|  | Sonipat | 79.4 |
|  | Jind | 84.3 |
|  | Fatehabad | 84.3 |
|  | Sirsa | 98.4 |
|  | Hisar | 88.9 |
|  | Bhiwani | 93.7 |
|  | Rohtak | 84.4 |
|  | Jhajjar | 84.3 |
|  | Mahendragarh | 70.6 |
|  | Rewari | 88.1 |
|  | Gurgaon | 79.1 |
|  | Mewat | 92.4 |
|  | Faridabad | 65.7 |
|  | Palwal | 86.2 |
|  |  |  |
| **Himachal Pradesh** | Chamba | 84.5 |
|  | Kangra | 76.8 |
|  | Lahul & Spiti | 80.2 |
|  | Kullu | 95.7 |
|  | Mandi | 75.6 |
|  | Hamirpur | 68.0 |
|  | Una | 73.3 |
|  | Bilaspur | 77.2 |
|  | Solan | 85.5 |
|  | Sirmaur | 85.7 |
|  | Shimla | 70.8 |
|  | Kinnaur | 87.4 |
|  |  |  |
| **Jammu & Kashmir** | Kupwara | 51.9 |
|  | Badgam | 66.1 |
|  | Punch | 50.3 |
|  | Rajouri | 54.9 |
|  | Kathua | 57.9 |
|  | Baramula | 56.1 |
|  | Bandipore | 62.2 |
|  | Srinagar | 67.9 |
|  | Ganderbal | 49.9 |
|  | Pulwama | 67.4 |
|  | Shupiyan | 64.3 |
|  | Anantnag | 62.9 |
|  | Kulgam | 56.3 |
|  | Doda | 42.1 |
|  | Ramban | 41.9 |
|  | Kishtwar | 46.6 |
|  | Udhampur | 72.0 |
|  | Reasi | 59.5 |
|  | Jammu | 49.4 |
|  | Samba | 51.3 |
|  |  |  |
| **Jharkhand** | Garhwa | 82.3 |
|  | Chatra | 81.0 |
|  | Kodarma | 86.2 |
|  | Giridih | 96.0 |
|  | Deoghar | 84.3 |
|  | Godda | 92.5 |
|  | Sahibganj | 86.7 |
|  | Pakur | 88.5 |
|  | Dhanbad | 88.0 |
|  | Bokaro | 89.2 |
|  | Lohardaga | 82.4 |
|  | Purbi Singhbhum | 80.7 |
|  | Palamu | 86.8 |
|  | Latehar | 84.5 |
|  | Hazaribagh | 91.1 |
|  | Ramgarh | 89.1 |
|  | Dumka | 77.9 |
|  | Jamtara | 86.4 |
|  | Ranchi | 88.8 |
|  | Khunti | 80.4 |
|  | Gumla | 78.0 |
|  | Simdega | 75.9 |
|  | Pashchimi Singhbhum | 94.2 |
|  | Saraikela Kharsawan | 79.2 |
|  |  |  |
| **Karnataka** | Belgaum | 92.4 |
|  | Bagalkot | 82.1 |
|  | Bijapur | 90.2 |
|  | Bidar | 84.6 |
|  | Raichur | 92.0 |
|  | Koppal | 85.3 |
|  | Gadag | 83.2 |
|  | Dharwad | 87.7 |
|  | Uttara Kannada | 67.5 |
|  | Haveri | 86.3 |
|  | Bellary | 84.8 |
|  | Chitradurga | 72.9 |
|  | Davanagere | 85.7 |
|  | Shimoga | 79.2 |
|  | Udupi | 62.9 |
|  | Chikmagalur | 75.9 |
|  | Tumkur | 81.7 |
|  | Bangalore | 74.3 |
|  | Mandya | 79.9 |
|  | Hassan | 81.7 |
|  | Dakshina Kannada | 60.5 |
|  | Kodagu | 78.2 |
|  | Mysore | 76.3 |
|  | Chamarajanagar | 73.0 |
|  | Gulbarga | 85.5 |
|  | Yadgir | 86.5 |
|  | Kolar | 86.8 |
|  | Chikkaballapura | 92.6 |
|  | Bangalore Rural | 81.0 |
|  | Ramanagara | 75.0 |
|  |  |  |
| **Kerala** | Kasaragod | 68.2 |
|  | Kannur | 61.7 |
|  | Wayanad | 70.2 |
|  | Kozhikode | 67.2 |
|  | Malappuram | 53.4 |
|  | Palakkad | 77.6 |
|  | Thrissur | 64.2 |
|  | Ernakulam | 70.7 |
|  | Idukki | 71.6 |
|  | Kottayam | 59.8 |
|  | Alappuzha | 80.6 |
|  | Pathanamthitta | 65.2 |
|  | Kollam | 54.4 |
|  | Thiruvananthapuram | 42.7 |
|  |  |  |
| **Lakshadweep** | Lakshadweep | 72.8 |
|  |  |  |
| **Madhya Pradesh** | Sheopur | 95.5 |
|  | Morena | 90.8 |
|  | Bhind | 93.0 |
|  | Gwalior | 95.9 |
|  | Gatia | 94.9 |
|  | Shivpuri | 86.3 |
|  | Tikamgarh | 87.1 |
|  | Chhatarpur | 88.8 |
|  | Panna | 81.4 |
|  | Sagar | 84.7 |
|  | Damoh | 86.1 |
|  | Satna | 88.3 |
|  | Rewa | 90.1 |
|  | Umaria | 83.0 |
|  | Neemuch | 81.0 |
|  | Mandsaur | 94.7 |
|  | Ratlam | 79.9 |
|  | Ujjain | 81.4 |
|  | Shajapur | 89.2 |
|  | Dewas | 82.7 |
|  | Dhar | 79.6 |
|  | Indore | 80.3 |
|  | Khargone (West Nimar) | 87.3 |
|  | Barwani | 82.0 |
|  | Rajgarh | 95.0 |
|  | Vidisha | 90.0 |
|  | Bhopal | 74.5 |
|  | Sehore | 92.4 |
|  | Raisen | 94.7 |
|  | Betul | 82.2 |
|  | Harda | 90.9 |
|  | Hoshangabad | 93.6 |
|  | Katni | 73.1 |
|  | Jabalpur | 88.6 |
|  | Narsimhapur | 77.1 |
|  | Dindori | 96.5 |
|  | Mandla | 92.7 |
|  | Chhindwara | 76.5 |
|  | Seoni | 77.4 |
|  | Balaghat | 81.1 |
|  | Guna | 94.3 |
|  | Ashoknagar | 89.6 |
|  | Shahdol | 84.5 |
|  | Anuppur | 77.7 |
|  | Sidhi | 90.5 |
|  | Singrauli | 81.6 |
|  | Jhabua | 86.2 |
|  | Alirajpur | 89.6 |
|  | Khandwa (East Nimar) | 93.2 |
|  | Burhanpur | 88.7 |
|  |  |  |
| **Maharashtra** | Nandurbar | 86.5 |
|  | Dhule | 71.1 |
|  | Jalgaon | 88.4 |
|  | Buldana | 90.4 |
|  | Akola | 86.1 |
|  | Washim | 73.9 |
|  | Amravati | 88.3 |
|  | Wardha | 85.5 |
|  | Nagpur | 87.4 |
|  | Bhandara | 96.4 |
|  | Gondiya | 89.0 |
|  | Gadchiroli | 80.7 |
|  | Chandrapur | 93.7 |
|  | Yavatmal | 61.2 |
|  | Nanded | 94.6 |
|  | Hingoli | 83.9 |
|  | Parbhani | 92.8 |
|  | Jalna | 88.2 |
|  | Aurangabad | 90.5 |
|  | Nashik | 83.9 |
|  | Thane | 77.2 |
|  | Mumbai Suburban | 67.4 |
|  | Mumbai | 83.9 |
|  | Raigarh | 76.3 |
|  | Pune | 71.5 |
|  | Ahmadnagar | 85.0 |
|  | Bid | 66.6 |
|  | Latur | 85.8 |
|  | Osmanabad | 80.7 |
|  | Solapur | 72.9 |
|  | Satara | 83.8 |
|  | Satnagiri | 74.0 |
|  | Sindhudurg | 58.4 |
|  | Kolhapur | 91.0 |
|  | Sangli | 79.9 |
|  |  |  |
| **Manipur** | Senapati | 78.9 |
|  | Tamenglong | 65.3 |
|  | Churachandpur | 73.6 |
|  | Bishnupur | 67.4 |
|  | Thoubal | 68.3 |
|  | Imphal West | 45.7 |
|  | Imphal East | 65.8 |
|  | Ukhrul | 68.4 |
|  | Chandel | 77.1 |
|  |  |  |
| **Meghalaya** | West Garo Hills | 54.9 |
|  | East Garo Hills | 34.9 |
|  | South Garo Hills | 21.6 |
|  | West Khasi Hills | 62.1 |
|  | Ribhoi | 53.4 |
|  | East Khasi Hills | 55.1 |
|  | Jaintia Hills | 64.4 |
|  |  |  |
| **Mizoram** | Mamit | 66.2 |
|  | Kolasib | 57.6 |
|  | Aizawl | 52.1 |
|  | Champhai | 62.6 |
|  | Serchhip | 50.1 |
|  | Lunglei | 64.6 |
|  | Lawngtlai | 75.7 |
|  | Saiha | 70.6 |
|  |  |  |
| **Nagaland** | Mon | 70.0 |
|  | Mokokchung | 83.2 |
|  | Zunheboto | 65.8 |
|  | Wokha | 53.7 |
|  | Dimapur | 59.7 |
|  | Phek | 59.0 |
|  | Tuensang | 89.0 |
|  | Longleng | 77.3 |
|  | Kiphire | 53.6 |
|  | Kohima | 52.2 |
|  | Peren | 71.8 |
|  |  |  |
| **Delhi** | Northwest | 78.2 |
|  | North | 89.1 |
|  | Northeast | 75.2 |
|  | East | 83.7 |
|  | New Delhi | 70.1 |
|  | Central | 83.8 |
|  | West | 88.7 |
|  | Southwest | 71.6 |
|  | South | 79.3 |
|  |  |  |
| **Odisha** | Bargarh | 75.4 |
|  | Jharsuguda | 77.0 |
|  | Sambalpur | 82.8 |
|  | Debagarh | 74.8 |
|  | Sundargarh | 81.0 |
|  | Kendujhar | 65.4 |
|  | Mayurbhanj | 73.8 |
|  | Baleshwar | 81.4 |
|  | Bhadrak | 73.7 |
|  | Kendrapara | 80.0 |
|  | Jagatsinghapur | 76.1 |
|  | Cuttack | 81.3 |
|  | Jajapur | 63.0 |
|  | Dhenkanal | 87.9 |
|  | Anugul | 85.5 |
|  | Nayagarh | 67.8 |
|  | Khordha | 72.7 |
|  | Puri | 74.7 |
|  | Ganjam | 80.5 |
|  | Gajapati | 88.2 |
|  | Kandhamal | 84.4 |
|  | Baudh | 87.7 |
|  | Subarnapur | 63.1 |
|  | Balangir | 87.8 |
|  | Nuapada | 91.8 |
|  | Kalahandi | 84.6 |
|  | Rayagada | 75.3 |
|  | Nabarangapur | 87.3 |
|  | Koraput | 90.9 |
|  | Malkangiri | 70.3 |
|  |  |  |
| **Puducherry** | Yanam | 48.7 |
|  | Puducherry | 47.0 |
|  | Mahe | 53.4 |
|  | Karaikal | 44.2 |
|  |  |  |
| **Punjab** | Gurdaspur | 95.9 |
|  | Kapurthala | 85.2 |
|  | Jalandhar | 83.9 |
|  | Hoshiarpur | 91.6 |
|  | Sangrur | 83.6 |
|  | Fatehgarh Sahib | 86.0 |
|  | Ludhiana | 92.4 |
|  | Moga | 86.1 |
|  | Firozpur | 86.5 |
|  | Muktsar | 87.5 |
|  | Faridkot | 84.7 |
|  | bathinda | 77.6 |
|  | Mansa | 85.3 |
|  | Patiala | 71.5 |
|  | Amritsar | 88.8 |
|  | Tarn Taran | 95.6 |
|  | Rupnagar | 89.5 |
|  | Sahibzada Ajit Singh | 90.8 |
|  | Shahid Bhagat Singh Nagar | 81.8 |
|  | Barnala | 64.3 |
|  |  |  |
| **Rajasthan** | Ganganagar | 96.0 |
|  | Hanumangarh | 96.2 |
|  | Bikaner | 94.8 |
|  | Churu | 84.7 |
|  | Jhunjhunun | 95.0 |
|  | Alwar | 89.9 |
|  | Bharatpur | 87.1 |
|  | Dhaulpur | 91.8 |
|  | Karauli | 94.6 |
|  | Sawai Madhopur | 91.9 |
|  | Dausa | 87.6 |
|  | Jaipur | 83.5 |
|  | Sikar | 87.6 |
|  | Nagaur | 91.9 |
|  | Jodhpur | 92.8 |
|  | Jaisalmer | 94.1 |
|  | Barmer | 95.7 |
|  | Jalor | 98.5 |
|  | Sirohi | 94.4 |
|  | Pali | 92.5 |
|  | Ajmer | 93.4 |
|  | Tonk | 93.4 |
|  | Bundi | 96.1 |
|  | Bhilwara | 93.3 |
|  | Rajsamand | 92.2 |
|  | Dungarpur | 97.9 |
|  | Banswara | 97.3 |
|  | Chittaurgarh | 97.3 |
|  | Kota | 90.4 |
|  | Baran | 95.9 |
|  | Jhalawar | 99.0 |
|  | Udaipur | 94.4 |
|  | Pratapgarh | 97.8 |
|  |  |  |
| **Sikkim** | North District | 49.2 |
|  | West District | 59.5 |
|  | South District | 56.4 |
|  | East District | 46.2 |
|  |  |  |
| **Tamil Nadu** | Thiruvallur | 53.3 |
|  | Chennai | 64.8 |
|  | Kancheepuram | 46.9 |
|  | Vellore | 49.4 |
|  | Tiruvannamalai | 40.6 |
|  | Viluppuram | 50.4 |
|  | Salem | 50.8 |
|  | Namakkal | 41.8 |
|  | Erode | 44.8 |
|  | The Nilgiris | 47.0 |
|  | Dindigul | 41.7 |
|  | Karur | 44.9 |
|  | Tiruchirappalli | 53.0 |
|  | Perambalur | 33.3 |
|  | Ariyalur | 53.3 |
|  | Cuddalore | 56.7 |
|  | Nagapattinam | 51.4 |
|  | Thiruvarur | 48.8 |
|  | Thanjavur | 40.3 |
|  | Pudukkottai | 45.7 |
|  | Sivaganga | 43.0 |
|  | Madurai | 49.8 |
|  | Theni | 54.1 |
|  | Virudhunagar | 42.0 |
|  | Ramanathapuram | 41.5 |
|  | Toothukkudi | 48.1 |
|  | Tirunelveli | 35.0 |
|  | Kanniyakumari | 31.9 |
|  | Dharmapuri | 58.5 |
|  | Krishnagiri | 54.7 |
|  | Coimbatore | 50.1 |
|  | Tiruppur | 51.7 |
|  |  |  |
| **Tripura** | West Tripura | 76.4 |
|  | South Tripura | 90.6 |
|  | Dhalai | 85.5 |
|  | North Tripura | 95.9 |
|  |  |  |
| **Uttar Pradesh** | Saharanpur | 92.4 |
|  | Muzaffarnagar | 90.0 |
|  | Bijnor | 92.7 |
|  | Moradabad | 89.7 |
|  | Rampur | 92.4 |
|  | Jyotiba Phule Nagar | 93.2 |
|  | Meerut | 85.8 |
|  | Baghpat | 96.2 |
|  | Ghaziabad | 96.7 |
|  | Gautam Buddha Nagar | 88.6 |
|  | Bulandshahr | 92.4 |
|  | Aligarh | 94.7 |
|  | Mahamaya Nagar | 87.0 |
|  | Mathura | 85.7 |
|  | Agra | 92.4 |
|  | Firozabad | 92.5 |
|  | Mainpuri | 89.5 |
|  | Badaun | 95.3 |
|  | Bareilly | 92.4 |
|  | Pilibhit | 94.8 |
|  | Shahjahanpur | 90.7 |
|  | Kheri | 89.2 |
|  | Sitapur | 90.7 |
|  | Hardoi | 85.2 |
|  | Unnao | 95.1 |
|  | Lucknow | 93.8 |
|  | Rae bareli | 93.9 |
|  | Farrukhabad | 91.8 |
|  | Kannauj | 89.4 |
|  | Etawah | 94.5 |
|  | Auraiya | 93.7 |
|  | Kanpur Dehat | 91.2 |
|  | Kanpur Nagar | 90.1 |
|  | Jalaun | 98.0 |
|  | Jhansi | 83.6 |
|  | Lalitpur | 87.5 |
|  | Hamirpur | 96.5 |
|  | Mahoba | 93.5 |
|  | Banda | 94.7 |
|  | Chitrakoot | 84.9 |
|  | Fatehpur | 93.9 |
|  | Pratapgarh | 98.2 |
|  | Kaushambi | 99.3 |
|  | Allahabad | 89.0 |
|  | Barabanki | 91.0 |
|  | Faizabad | 96.5 |
|  | Ambedkar Nagar | 91.2 |
|  | Sultanpur | 92.1 |
|  | Bahraich | 90.5 |
|  | Shrawasti | 85.3 |
|  | Balrampur | 82.9 |
|  | Gonda | 88.2 |
|  | Siddharth Nagar | 94.0 |
|  | Basti | 94.2 |
|  | Sant Kabir Nagar | 91.2 |
|  | Mahrajganj | 82.5 |
|  | Gorakhpur | 90.7 |
|  | Kushinagar | 81.4 |
|  | Deoria | 93.7 |
|  | Azamgarh | 91.5 |
|  | Mau | 88.4 |
|  | Ballia | 89.6 |
|  | Jaunpur | 90.3 |
|  | Ghazipur | 94.8 |
|  | Chandauli | 86.8 |
|  | Varanasi | 92.5 |
|  | Sant ravidas nagar (bhadohi) | 87.5 |
|  | Mirzapur | 87.9 |
|  | Sonbhadra | 93.6 |
|  | Etah | 95.5 |
|  | Kanshiram Nagar | 93.7 |
|  |  |  |
| **Uttarakhand** | Uttarkashi | 87.6 |
|  | Chamoli | 82.7 |
|  | Rudraprayag | 79.5 |
|  | Tehri Garhwal | 87.4 |
|  | Dehradun | 78.1 |
|  | Garhwal | 90.3 |
|  | Pithoragarh | 73.5 |
|  | Bageshwar | 79.0 |
|  | Almora | 82.6 |
|  | Champawat | 73.5 |
|  | Nainital | 76.7 |
|  | Udham Singh Nagar | 77.9 |
|  | Hardwar | 88.1 |
|  |  |  |
| **West Bengal** | Darjiling | 74.3 |
|  | Jalpaiguri | 57.8 |
|  | Kochbihar | 53.5 |
|  | Uttar Dinajpur | 69.6 |
|  | Dakshin Dinajpur | 65.0 |
|  | Maldah | 83.2 |
|  | Murshidabad | 70.1 |
|  | Birbhum | 67.9 |
|  | Barddhaman | 57.6 |
|  | Nadia | 47.5 |
|  | North Twenty-Four Parganas | 63.7 |
|  | Hugli | 61.7 |
|  | Bankura | 55.9 |
|  | Puruliya | 67.9 |
|  | Haora | 66.7 |
|  | Kolkata | 78.4 |
|  | South Twenty-Four Parganas | 58.5 |
|  | Paschim Medinipur | 60.9 |
|  | Purba Medinipur | 61.4 |
|  |  |  |
| **Telangana** | Adilabad | 82.5 |
|  | Nizamabad | 82.7 |
|  | Karimnagar | 77.4 |
|  | Medak | 81.3 |
|  | Hyderabad | 57.2 |
|  | Rangareddy | 80.5 |
|  | Mahbubnagar | 86.4 |
|  | Nalgonda | 77.8 |
|  | Warangal | 76.3 |
|  | Khammam | 77.9 |
|  |  |  |
| **Ladakh** | Leh | 59.6 |
|  | Kargil | 57.3 |
| *Note*  District and corresponding shaded estimates are the Aspirational Districts of India. | | |
